# JNK1 controls dendritic field size in L2/3 and L5 of the motor cortex, constrains soma size, and influences fine motor coordination

**DOI:** 10.3389/fncel.2014.00272

**Published:** 2014-09-12

**Authors:** Emilia Komulainen, Justyna Zdrojewska, Erika Freemantle, Hasan Mohammad, Natalia Kulesskaya, Prasannakumar Deshpande, Francesca Marchisella, Raghavendra Mysore, Patrik Hollos, Kimmo A. Michelsen, Mats Mågard, Heikki Rauvala, Peter James, Eleanor T. Coffey

**Affiliations:** ^1^Turku Centre for Biotechnology, Åbo Akademi University and University of TurkuTurku, Finland; ^2^Neuroscience Center, University of HelsinkiHelsinki, Finland; ^3^Department of Biosciences, Åbo Akademi UniversityTurku, Finland; ^4^Institute for Immune Technology, Medicon Village, University of LundLund, Sweden

**Keywords:** JNK, dendrite, motor cortex, MAP2, microtubules, cytoskeleton, schizophrenia, behavior

## Abstract

Genetic anomalies on the JNK pathway confer susceptibility to autism spectrum disorders, schizophrenia, and intellectual disability. The mechanism whereby a gain or loss of function in JNK signaling predisposes to these prevalent dendrite disorders, with associated motor dysfunction, remains unclear. Here we find that JNK1 regulates the dendritic field of L2/3 and L5 pyramidal neurons of the mouse motor cortex (M1), the main excitatory pathway controlling voluntary movement. In *Jnk1-/-* mice, basal dendrite branching of L5 pyramidal neurons is increased in M1, as is cell soma size, whereas in L2/3, dendritic arborization is decreased. We show that JNK1 phosphorylates rat HMW-MAP2 on T1619, T1622, and T1625 (Uniprot P15146) corresponding to mouse T1617, T1620, T1623, to create a binding motif, that is critical for MAP2 interaction with and stabilization of microtubules, and dendrite growth control. Targeted expression in M1 of GFP-HMW-MAP2 that is pseudo-phosphorylated on T1619, T1622, and T1625 increases dendrite complexity in L2/3 indicating that JNK1 phosphorylation of HMW-MAP2 regulates the dendritic field. Consistent with the morphological changes observed in L2/3 and L5, *Jnk1-/-* mice exhibit deficits in limb placement and motor coordination, while stride length is reduced in older animals. In summary, JNK1 phosphorylates HMW-MAP2 to increase its stabilization of microtubules while at the same time controlling dendritic fields in the main excitatory pathway of M1. Moreover, JNK1 contributes to normal functioning of fine motor coordination. We report for the first time, a quantitative Sholl analysis of dendrite architecture, and of motor behavior in *Jnk1-/-* mice. Our results illustrate the molecular and behavioral consequences of interrupted JNK1 signaling and provide new ground for mechanistic understanding of those prevalent neuropyschiatric disorders where genetic disruption of the JNK pathway is central.

## INTRODUCTION

Dendrites are highly specialized, excitable compartments of neurons that are molecularly and functionally distinct from the axon. They receive and compute synaptic input and fine-tune the character of the action potential output (Williams and Stuart, 2003; London and Häusser, 2005). Notably, the computational properties of dendrites are directly influenced by the shape and extent of dendritic trees (Rall, 1977; Jaffe and Carnevale, 1999; London and Häusser, 2005; Spruston, 2008). Genetic disorders that involve well characterized anomalies in dendrite architecture include mental retardation syndromes Down’s, RETT, and fragile-X, where decreased arborization is common (Kaufmann and Moser, 2000; Zoghbi, 2003; Pardo and Eberhart, 2007; Jan and Jan, 2010). Conversely, autism and schizophrenia are associated with overgrowth of dendrites during development. Although these are highly heritable disorders, the genetic basis remains largely unknown, though large-scale genomic studies are starting to reveal candidates (Hyman, 2008; Fromer et al., 2014; Purcell et al., 2014).

c-Jun N-terminal kinase-1 (JNK1) has been implicated in the regulation of dendrite arborization in isolated neurons. For example, in cerebellar granule neurons JNK inhibition increases dendrite complexity (Björkblom et al., 2005), while in cortical and hippocampal neurons, JNK inhibitors reduce dendrite growth (Rosso et al., 2005; Podkowa et al., 2010). Yet, mechanistic details are lacking, and detailed quantitation of dendrite architecture using Sholl analysis in *Jnk* knockout mice has not been reported. Interestingly however, recent human genetics studies report deregulation of JNK pathway genes in several dendrite disorders. For example, JNK1 activity in the cortex is dependent on a kinase located on chromosome 16p11.2, a gene susceptibility *locus* for autism and schizophrenia (Weiss et al., 2008; McCarthy et al., 2009). Genetic risk for schizophrenia is associated with the JNK pathway (Winchester et al., 2012), and the *interleukin-1 receptor accessory protein like-1* gene, implicated in monogenic forms of mental retardation and autism, signals through JNK (Pavlowsky et al., 2010). Furthermore, chromosomal translocations leading to loss of function truncations of *JNK3* are associated with intellectual disability (Shoichet et al., 2006; Baptista et al., 2008; Kunde et al., 2013). These findings suggest that disruption of normal JNK function may be central to neuropsychiatric disorders that share irregularities in dendrite shape as a common hallmark.

To gain molecular understanding of the structural changes that occur in the brain upon disruption of JNK signaling, we set out to precisely characterize the dendrite architecture in *Jnk1-/-* mice using three-dimensional Sholl analysis. We generated phosphorylation site mutants of HMW-MAP2, a major substrate of JNK1 in dendrites (Kyriakis et al., 1995; Chang et al., 2003; Björkblom et al., 2005), and examined whether site-specific phosphorylation of HMW-MAP2 by JNK1 altered dendrite shape and microtubule integrity. Our findings show that JNK1 phosphorylates HMW-MAP2 on specific residues in the C-terminal domain to create a microtubule binding motif, leading to increased microtubule stabilization. Moreover, our investigation revealed significant structural alterations in L2/3 and L5 dendrites in the primary motor cortex of *Jnk1-/-* mice. Consistent with these findings, ectopic expression of GFP-HMW-MAP2^T1619D,T1622D,T1625D^ alone was sufficient to dramatically increase pyramidal neuron dendrite length and complexity in the motor cortex suggesting that phosphorylation of HMW-MAP2 on these residues has a major impact on dendritic field. Finally we show that the behavioral consequence of disrupted JNK1 signaling is impaired motor function.

## MATERIALS AND METHODS

### ANTIBODIES

Anti-MAP2 (AP20) recognizing HMW-MAP2 was from Leinco Technologies (St. Louis, MO, USA), Phospho-MAP2 (cat. no. 4544; RRID: AB_2144157) recognizing HMW-MAP2, and PJNK-Thr183/Tyr185 (cat. no. 9255S; RRID: AB_2235013) were from Cell Signaling Technology Inc. (Danvers, MA, USA) or from Biosource (cat. no. 44-682G; RRID: AB_1502039). JNK1 (clone no. G151-333; RRID: AB_399158) was from PharMingen (San Diego, CA, USA). Mouse anti-GFP (cat. no. JL-8; RRID: AB_10013427) was from Clontech (Mountain View, CA, USA). Anti-mouse β-tubulin (cat. no. KMX-1; RRID: AB_94650) was from Chemicon (Temecula, CA, USA) and anti-ankyrin-G was from NeuroMab (clone N106/36; cat. no. 75-146; RRID: AB_10673030.

### PLASMIDS

pEGFP-HMW-MAP2 and pEGFP-NES-Jun were previously described (Björkblom et al., 2005; Tararuk et al., 2006). Phosphorylation site mutants of HMW-MAP2, EGFP-HMW-MAP2^T1619A/T1622A/T1625A^ (abbreviated to GFP-MAP2-AAA) and EGFP-MAP2^T1619D/T1622D/T1625D^ (abbreviated to GFP-MAP2-DDD), were prepared by insertional overlapping PCR using mutagenic primers as previously described (Hongisto et al., 2008). The phosphorylation site numbering is based on the rat HMW-MAP2 Uniprot entry P15146. For *in utero* electroporation, the CMV promoter in EGFP-HMW-MAP2^WT^ and EGFP-HMW-MAP2^T1619D/T1622D/T1625D^ was changed to a CAG promoter for optimal expression in brain. MAP2C was isolated by PCR from rat brain cDNA. It was inserted downstream of GST in the pGEX-6P3 vector (GE Healthcare) using the pGEMTe cloning vector (Promega). pCDNA3-MKK7-JNK1 was a gift from Roger J. Davis (HHMI, Worcester, MA, USA).

### PHOSPHORYLATION

GST-MAP2 0.1–0.4 μM was phosphorylated with active GST-JNK1α1 or GST-JNK3α1, of comparable specific activities, in 30 μl kinase buffer (10 mM PBS pH 7.4, 2 mM EGTA, 1 mM DTT, 10 mM MgCl2, 0.1% v/v Triton X100) supplemented with 5 μCi of [γ^32^P]-ATP and 25 μM non-isotopically labeled ATP. The reaction was carried out for 1 h at 30°C and stopped by the addition of 4× Laemmli sample buffer. GST-cJun(5–89) at 2.22 μM concentration was used as a positive control to monitor the catalytic activities of JNK1α1 and JNK3α1. Samples were resolved by SDS–PAGE, stained with Coomassie brilliant blue, destained and analyzed by autoradiography and phosphorimaging. Velocity was calculated using Michaelis–Menten kinetics and was plotted against substrate concentration. For phosphosite analysis, the phosphorylated bands corresponding to full length GST-MAP2 were excised from the gel and subjected to in gel digestion and mass spectrometry analysis as described below. Metabolic labeling of COS-7 cells using [γ-^32^P]-ATP was carried out as previously (Bjorkblom et al., 2012).

### TISSUE EXTRACT PREPARATION

Forebrains from mice at postnatal day 0 or 2 were rapidly extracted after decapitation and post-translational modifications were preserved using a heat stabilizer (Denator, Sweden). Frozen tissues were homogenized using an Ultra Turrax homogenizer in ice-cold *lysis buffer* (20 mm HEPES, pH 7.4, 2 mm EGTA, 50 mm β-glycerophosphate, 1 mm dithiothreitol, 1 mm Na_3_VO_4_, 1% Triton X-100, 10% glycerol, 1 mm benzamidine, 50 mm NaF, 1 μg/ml leupeptin, 1 μg/ml, pepstatin, 1 μg/ml aprotinin, and 100 μg/ml PMSF). Lysates were normalized for protein using the Bradford method before SDS-PAGE.

### IN-GEL DIGESTION AND PHOSPHOPEPTIDE ENRICHMENT

*In vitro* phosphorylated proteins were separated on 12% Criterion gels (Bio-Rad Laboratories, Hercules, CA, USA), gels were washed in Milli-Q water, stained 1 h with GelCode (Thermo Scientific, Rockford, IL, USA), destained overnight in Milli-Q water. Each lane was manually sliced into five fractions and slices were destained then reduced and alkylated before digestion with 12.5 μg/ml sequencing grade modified porcine trypsin (Promega, Madison, WI, USA) overnight at 37°C as previously described (Bjorkblom et al., 2012). Peptides were eluted in 75% ACN, 1% FA. 60 μl of peptides were dried and immediately subjected to phospho-peptide enrichment. The peptides (ca. 50 μg/sample) were re-suspended in 150 μl binding buffer (1 M glycolic acid, 80% ACN, 5% TFA) and mixed with 50 μl homogenous suspension of TiO_2_ magnetic sepharose beads (GE Healthcare Bio-Science AB, Uppsala, Sweden) that had previously been washed five times in the same buffer. Peptides were equilibrated with the beads, binding for 60 min at RT with gentle rocking. The beads were washed three times with 200 μl washing Buffer (80% ACN, 1% TFA) and peptides were eluted twice adding in total 100 μl 5% NH_3_ pH 12. The pH of the solutions was lowered to <3 adding 5 μl 88% FA prior to sample clean up using C18 UltraMicroSpin columns (The Nest Group Inc., Southboro, MA, USA). Eluted peptides were then dried in a Speedvac, resuspended in 0.1% FA and then immediately analyzed by LC–MS.

### IMMUNOBLOT ANALYSIS AND QUANTIFICATION

Cells were stimulated as indicated, washed in PBS, and lysed with *Laemmli* sample buffer. Samples were resolved on 5% (MAP2) or 10% SDS-PAGE and transferred by semi-dry transfer to nitrocellulose. Blots were developed using the enhanced chemiluminescence detection method. Films were pre-flashed, and non-saturated exposures were digitized by flatbed scanning and quantified by densitometry.

### CELL CULTURE AND TRANSFECTION

Mouse embryonic fibroblasts (MEFs) were cultured in minimal essential medium (MEM) supplemented with 10% (v/v) bovine calf serum, 2 mM glutamine, 50 U/ml penicillin, 50 μg/ml streptomycin, and non-essential amino acids (Sigma) were added. COS-7 cells were cultured in MEM supplemented with 10% (v/v) bovine calf serum, 2 mM glutamine, 50 U/ml penicillin, and 50 μg/ml streptomycin. All cells were grown with 5% CO_2_ at 37°C. Cell lines were transfected with Lipofectamine-2000 (Invitrogen, Carlsbad, CA, USA) according to manufacturer’s instructions. Cell process measurements were performed as described previously (Björkblom et al., 2005).

### IMMUNOSTAINING

Hippocampal neurons at 12 days *in vitro* were fixed with 4% paraformaldehyde for 20 min and permeabilized for 3 min with 1% TX100 in phosphate-buffered saline (PBS). Following block in 10% fetal calf serum, cells were stained using anti-JNK1 (Pharmingen) at 1:100, ankyrin-G (NeuroMab) at 1:800, or P-JNK (Biosource) at 1:200 and detected with Alexa-488/568 conjugated secondary antibodies at 1:500.

### MEASUREMENT OF TUBULIN AND HMW-MAP2 POLYMERIZATION

MEF2 cells were transfected with 50% Venus-tubulin and 50% EGFP-HMW-MAP2 variants. After 24 h the cells were washed with pre-warmed *PEM buffer* (80 mM pipes, 1 mM MgCl_2_, 2 mM EGTA, pH 6.8, PMSF, 1 μg/ml aprotonin, 1 μg/ml pepstatin, 1 μg/ml leupeptin). Cells were then lysed in +37°C *PEM buffer* containing 0.15% TX-100. After 1 min lysis, the liquid phase was pipetted from the dish and collected as the soluble fraction. The TX-100 insoluble fraction was collected in laemmli buffer. Fractions were analyzed by immunoblotting and quantified using densitometry.

### LUCIFER YELLOW LOADING

Mice at 6–8 months were anesthetized with 0.3 mg/g Pentobarbital (Mebunat 60 mg/ml) mixed 50:50 with 0.9% NaCl. Mice were perfused transcardially on ice bedding using 10–20 ml 0.9% NaCl followed by 25–50 ml of buffer comprising 4% paraformaldehyde and 0.125% glutaraldehyde in 0.1 M *Sorensen’s phosphate buffer* (NaH_2_PO_4_–Na_2_HPO_4_, pH 7.2). The brain was post-fixed in 50 ml 4.0% PFA in phosphate buffer for 4–12 h at +4°C. Coronal sections of 180–200 μm were sectioned using a vibratome. The nuclei were visualized using DAPI (4^′^,6-diamino-2-phenylindole, dihydrochloride (Invitrogen). Pyramidal neurons in the motor cortex and the hippocampus were located according to the Atlas of C57BL/6 mouse brains (Hof et al., 2000). Selected neurons were injected by iontophoresis with lucifer yellow dye (Invitrogen) using pulled borosilicate glass tubes (World Precision Instruments). The DC current source was 2–6 nA from a dual micro-iontophoresis current generator, model 260 (World Precision Instruments). After dye loading, brain slices were transferred to a slide and mounted using Shandon PermaFluor mounting medium (ThermoFisher).

### *IN UTERO* ELECTROPORATION

For dendrite analysis timed-pregnant mice C57/B6 (WT and *Jnk1-/-*) carrying E15 embryos were anesthetized with isoflurane (induction, 4%; surgery, 2.0%). Following anaesthetization pre-emptive analgesia was administered subcutaneously with a dose of buprenorphine 0.05–1 mg/kg (Temgesic® from Schering-Plow), and the uterine horns were exposed by laparotomy. GFP-CAG-HMW-MAP2^WT^ and GFP-CAG-HMW- MAP2^T1619D,T1622D,T1625D^ (2 μl) containing 0.8% Fast-Green (wt/vol) was injected into the lateral ventricles of embryos. After soaking the uterine horn with a PBS solution, the embryo’s head was carefully held between tweezer electrodes and DNA was electroporated using a CUY21E square wave electroporator (NEPA Gene). Electrical pulses (45 V, 50 ms) were passed five times at 1 s intervals. Uteri were returned to the abdominal cavity and embryos allowed to develop normally until delivery. Animals were sacrificed at P21. The procedures were approved by the National Animal Experimental Board. Collected tissues were fixed in 4% paraformaldehyde for 24 h, immersed in 30% sucrose (wt/vol) and frozen using isopentane. Cryosections (50 μm) were cut coronally and z-stack images were collected using a Zeiss LSM 780 confocal microscope with 20× air objective. LSM images for dendrite analysis collected from M1 were acquired following “The Mouse Brain in Stereotactic Coordinates, Third edition” by Franklin K.B.J and Paxinos G. Coronal sections selected for analysis were from the region between 5.78 and 4.42 mm (interaural) with respect to bregma 1.98–0.62 mm. The anterior forceps of the corpus callosum, anterior commisure, and the lateral ventricle was used as a landmark to recognize region of interest. Further dendrite analysis was done with Neurolucida software.

### THREE-DIMENSIONAL IMAGING AND SHOLL ANALYSIS

For lucifer yellow loaded cells, 1 μm z sections spanning 40–80 μm were acquired using a Leica TCS SP2 microscope with 20× objective. Spiny neurons with large Hoescht-33342-stained nuclei and pyramidal morphology were selected for analysis. Basal dendrites were traced manually and subjected to Sholl analysis using Neurolucida software (MBF, Bioscience). Fine apical dendrites were excluded from the analysis as complete loading including tufts was achieved in too few cells. Scoring was performed blind. One-way ANOVA was used to determine significant differences among data sets. For analysis of *in utero* elctroporated mice, confocal sections 0.7-μm thick were acquired using the Zeiss LSM-780 microscope and a 20× objective. Sections were reconstructed manually using Neurolucida software (MBF, Bioscience). Neurons with sufficient GFP signal to detect higher order branching were selected for analysis. Branched structure analysis was performed for each tree. Sholl analysis was performed for intersections using 10 μm concentric circles surrounding the cell soma. For soma size analysis, cells with a clear beginning and end point in the *z*-plane were traced for each stack and cross sectional area and perimeter were evaluated using Neurolucida. One-way ANOVA was used to determine significant differences for the Sholl intersections while *t*-test was used for branch order and soma size measures. Statistical analysis was performed in Graphpad Prism (version 5). For all the dendrite analysis, pyramidal cells were pooled into one group, as different pyramidal cells could not be accurately distinguished without full labeling of apical dendrites. Despite this, the difference between genotypes greatly exceeded the differences seen in dendrite complexity that exists for L5 neuronal subtypes (Oswald et al., 2013). The same applies to the changes in soma size.

### MOUSE BEHAVIOR

For behavior assessment, male mice were group-housed in standard cages with bedding and nesting material under a 12 h light–dark cycle. Food and water were provided *ad libitum*. Behavior assessment was started when animals were 8–9 weeks old (beam, rotarod, and suspended wire) and 3 or 7 months old (footprint test). Experiments were performed with 1–2 day intervals between tests. Experiments were carried out between 10:00 and 15:00 h. For all tests (with the exception of the footprint test), video-tracking was used. Scoring was carried out using video and sound material by an experimenter that was blinded to the genotype.

### BEAM

Coordination and motor ability was assessed. A mouse was placed in the middle of a horizontal beam (2 cm diameter, 120 cm length) raised 40 cm. Color painted marks divided the beam into 10 cm intervals. Mice were allowed to explore beam for 2 min or until they fell from the beam. Time spent on the beam and the number of crossed marks were calculated. After 1 h interval a second trial was repeated. Eighteen male mice (8 *Jnk1-/-* and 10 wild-type littermates) were used.

### ROTA-ROD

Evaluation of motor ability and motor learning was performed on the rotarod (Ugo Basile, Italy) on 2 days. Every experimental day included three trials with 1 h interval. The mouse was placed on the rotating drum with acceleration (from 4 to 40 r.p.m. over 5 min). The latency to fall (6 min cut-off time) was measured over all sessions. Eighteen male mice (8 *Jnk1-/-* and 10 wild-type littermates) were used.

### SUSPENDED BAR TEST

Mice were placed hanging from their forepaws on the center of a wire coat hanger (diameter 3 mm, length 39 cm) at a height of 41 cm above a container with soft bedding. After one training period, mice were video tracked for 45 s. Afterwards separate latencies were scored; (1) time to finish was the time taken to assume an upright position and reach the corner of the coat-hanger with the forepaws, (2) time to get “up” was the time taken to climb further onto the diagonal bar and touch 5 cm along with the forepaws, and (3) time to fall from the wire. A penalty score of 45 s was added to the “finish” score for mice that fell from the bar or failed to complete the task. A penalty of 45 s was added to the “up” score of mice that failed to reach 5 cm along the diagonal bar.

### INVERTED GRID TEST

Mice were placed on a metal grid (spacing 1 cm^2^) and allowed to grip the grid with four paws. The grid was inverted at an angle of 180° 10 cm above the ground and the time for the mouse to fall onto soft bedding was measured in seconds. The monitoring time was 2 min.

### FOOTPRINT TEST

The footprint analysis was performed with 12 wild-type and 12 *Jnk1-/-* mice at 3 months or with five wild-type and seven *Jnk1-/-* mice at 7 months. The hind paws of mice were coated with ink and mice were encouraged to walk along a runway with side walls of 10 cm. The runway was covered with paper that was 7.5 cm wide, and 80 cm long. Footprints were used to measure a series of 6–10 sequential steps recorded in three trials per mouse. Stride length was measured between the central pads of two consecutive prints on each side. Stride width was measured between the central pads of two footprints, one on either side. Three trials per mouse were meaned. SEMs are shown.

### ANATOMICAL MEASUREMENTS

Body weight of mice at 8- to 9-weeks old was measured before and after the battery of behavioral tests [beam, rotarod, acoustic startle, water-maze (not shown)]. The body weight of 3- and 7-month-old animals was measured before the footprint test and the suspended bar tests were performed. To measure hindlimb and forelimb length, mice after behavioral tests were sacrificed and pinned to a polystyrene platform. The joint between the forelimb and the clavicle was determined by examination. The distance from this joint to the end of the paw was measured. For measurement of hindlimb, the joint between the hindlimb and the hip was felt by examination with fingers. The distance from this join to the tip of the paw was measured. Distance between the forelimbs and hindlimbs was measured in a similar way according to the scheme.

### STATISTICAL ANALYSIS

Testing of sample variances was done by Student’s *t*-test or by one-way ANOVA followed by Bonferroni *post hoc* test using QI Macros SPC software (KnowWare International, Inc.).

## RESULTS

### JNK1 PHOSPHORYLATES RAT HMW-MAP2 ON T1619, T1622, AND T1625 IN THE PROLINE RICH DOMAIN (PRD)

**FIGURE 1:**
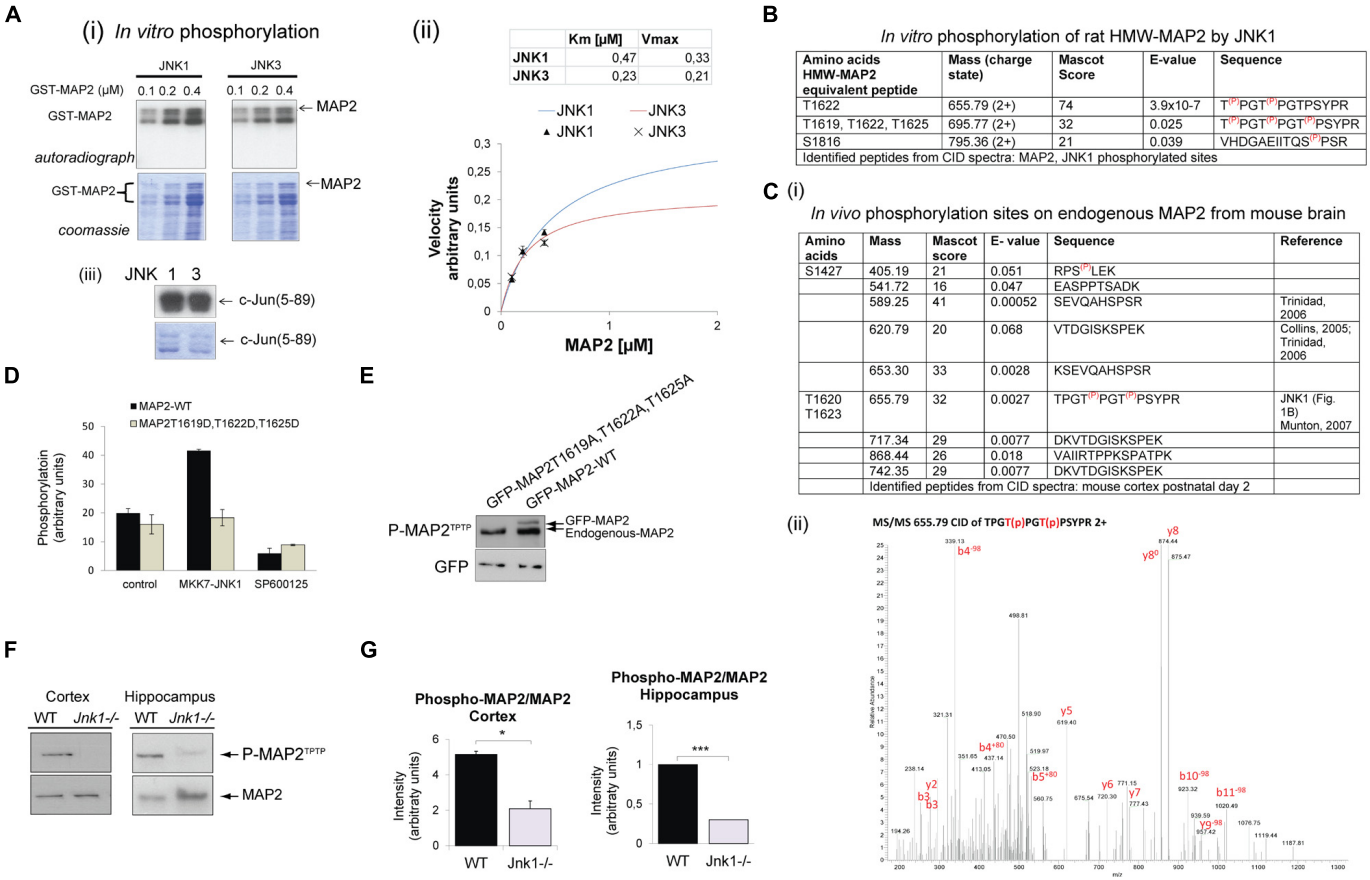
**JNK1 phosphorylates the PRD of MAP2 on T1619, T1622 and T1625 *in vivo* in the cortex and hippocampus. (A) (i)** Increasing concentrations of recombinant GST-MAP2 was phosphorylated *in vitro* by active recombinant JNK1α1 or JNK3α1. The relative phosphorylation by JNK1 and JNK3 can be visualized from the autoradiograph. **(ii)** Quantitative data obtained from phosphor-imaging is shown. JNK1 shows a higher *V*_max_ compared to JNK3. Meaned data ± SEM from four repeats is shown. **(iii)** The catalytic activities of JNK1 and JNK3 were tested for c-Jun(5–89) phosphorylation demonstrating comparable catalytic activity. **(B)** GST-MAP2 phosphorylated *in vitro* by JNK1 was subjected to phospho-peptide purification using TiO_2_ and MS/MS analysis. Identified peptides from collision induced dispersion spectra are shown in the table. T1622 and T1625 (HMW-MAP2 equivalent residues) were clearly phosphorylated. In addition a triply phosphorylated peptide phosphorylated on T1619, T1622, T1625 was identified. **(C) (i)** The *in vivo* phosphorylation sites of MAP2 determined from postnatal day 2 mouse cortex *ex vivo* are shown. Sites S1427, T1622, and T1625 contained covalently bound phosphate. (**ii**) A MS/MS spectra of the dual phosphorylated HMW-MAP2 phosphopeptide isolated from brain is shown. **(D)** To determine whether active JNK phosphorylated MAP2 in living cells, COS-7 cells were transfected with an active JNK chimera (MKK7-JNK1) together with GFP-MAP2^WT^ or GFP-MAP2^T1619D,T1622D,T1625D^ (as shown) and metabolically labeled with ^32^P-ATP. GFP-MAP2^WT^ phosphorylation increased in cells expressing MKK7-JNK1, but phosphorylation of GFP-MAP2^T1619,T1620D,T1620D^ did not. Addition of the JNK inhibitor SP600125 (10 μM) prevented the increase in GFP-MAP2^WT^ phosphorylation. Meaned data ± standard deviations are shown. **(E)** To validate the phospho-specificity of a phospho-MAP2 (P-MAP2^TPTP^) antibody generated against phosphorylated MAP2, GFP-MAP2^T1619A,T1622A,T1625A^, or GFP-MAP2^WT^ were expressed in neurons and immunoblotted. The P-MAP2^TPTP^ antibody recognized endogenous MAP2 as well as ectopically expressed GFP-MAP2^WT^, indicating that they are at least partly phosphorylated under basal conditions. The antibody did not detect GFP-MAP2^T1619A,T1622A,T1625A^ indicating specificity for the sites. **(F)** Tissue homogenates from cortex and hippocampi during the first postnatal week. Wild-type (WT) and *Jnk1-/-* mice were normalized for protein and immunoblotted for MAP2 or P-MAP2^TPTP^. **(G)** Quantitative data from blotting of post-natal cortex and hippocampus. P-MAP2^TPTP^ levels were normalized to MAP2. Means ± SEMs are shown from three repeats. *p < 0.05; ***p < 0.005.

Earlier work from our group and others showed that HMW-MAP2 is phosphorylated by JNK1 *in vitro* (Chang et al., 2003; Björkblom et al., 2005). To determine whether this phosphorylation was specific for JNK1, and to identify which amino acids are phosphorylated, we purified recombinant GST-MAP2 from *E. coli* and phosphorylated it *in vitro*. JNK1 and JNK3 both phosphorylated GST-MAP2, while JNK1 was slightly more efficient (**Figure 1A**), suggesting that either JNK isoform can phosphorylate MAP2 *in vivo*. To identify the phosphorylation sites on GST-MAP2, we carried out MS/MS analysis of TiO_2_-enriched phosphopeptides from the JNK1 phosphorylated GST-MAP2. This revealed three JNK1-phosphorylated sites in the C-terminal domain; T1619, T1622, and T1625 of rat HMW-MAP2 (**Figure 1B**).

Since JNK1 is constitutively active in developing brain (Coffey et al., 2000; Tararuk et al., 2006), we investigated whether any of the JNK1 sites on HMW-MAP2 were constitutively phosphorylated in mouse brain under physiological conditions. MS/MS analysis of phospho-peptides from postnatal day 2 brain identified 10 sites with basal phosphate occupancy (**Figure 1C**). Among these, the JNK1 sites T1620 and T1623 of mouse HMW-MAP2 were clearly phosphorylated. The MS/MS spectrum for the dual phosphorylated peptide is shown (**Figure 1C**). Mouse HMW-MAP2-T1617, -T1620, and -T1623 (Uniprot P20357) correspond to rat HMW-MAP2 sites -T1619, -1622, and -1625 (Uniprot P15146). Nine additional phosphopeptides were identified, among these four were previously described (Collins et al., 2005; Trinidad et al., 2006; Munton et al., 2007), while the remaining phosphopeptides were novel.

HMW-MAP2 encodes 43 (Pro)-X-Ser/Thr-Pro motifs (Sánchez et al., 1995) – putative motifs for JNK phosphorylation. As our analysis of JNK sites on MAP2 were carried out using GST-MAP2 (**Figure 1A**), which lacks the central domain (∼1360 amino acids) of high-molecular-weight (HMW) MAP2, we wanted to determine whether additional JNK sites could exist in the central domain. To test this, we performed metabolic labeling using GFP-HMW-MAP2 phospho-site mutants (**Figure 1D**). GFP-HMW-MAP2^WT^ or GFP-HMW-MAP2^T1619D,T1622D,T1625D^, where the JNK1 phosphorylation sites were mutated from threonine to aspartate, were expressed in COS-7 cells. Metabolic labeling of ATP pools with [γ-^32^P]-ATP allowed us to measure phosphate incorporation. GFP-MAP2^WT^ phosphorylation was increased upon co-expression of the constitutively active JNK1 chimera, MKK7-JNK1, while GFP-HMW-MAP2-^T1619D,T1622D,T1625D^ phosphorylation did not increase (**Figure 1D**), indicating that no additional JNK1 sites were present. Inhibition of JNK with the small molecule inhibitor SP600125 prevented JNK1-dependent phosphorylation increase.

### JNK1 PHOSPHORYLATES HMW-MAP2 ON EQUIVALENT SITES IN MOUSE BRAIN

To test whether JNK1 phosphorylated HMW-MAP2 on these sites *in vivo*, we utilized a commercial antibody that detects the phosphorylated PRD of HMW-MAP2. We examined the site specificity of this antibody, and found that it recognized GFP-HMW-MAP2^WT^ but not GFP-HMW-MAP2^T1619A,T1622A,T1625A^ (**Figure 1E**) indicating that it was specific for the JNK1-targeted threonine residues of HMW-MAP2 in a phosphorylated state. We used this antibody to compare the phosphorylation of endogenous HMW-MAP2 from wild-type and *Jnk1-/-* mice. Phospho-MAP2 levels were decreased in the cortex and hippocampus in *Jnk1-/-* mice, indicating that JNK1 phosphorylates these sites in immature brain (**Figures 1F,G**).

### GFP-MAP2^**T1619D,T1622D,T1625D**^ EXPRESSING CELLS EXTEND A GREATER NUMBER OF PROTRUSIONS, WHILE MAP2^**T1619A,T1622A,T1625A**^-EXPRESSING CELLS COMPLETELY FAIL TO GENERATE PROTRUSIONS

**FIGURE 2:**
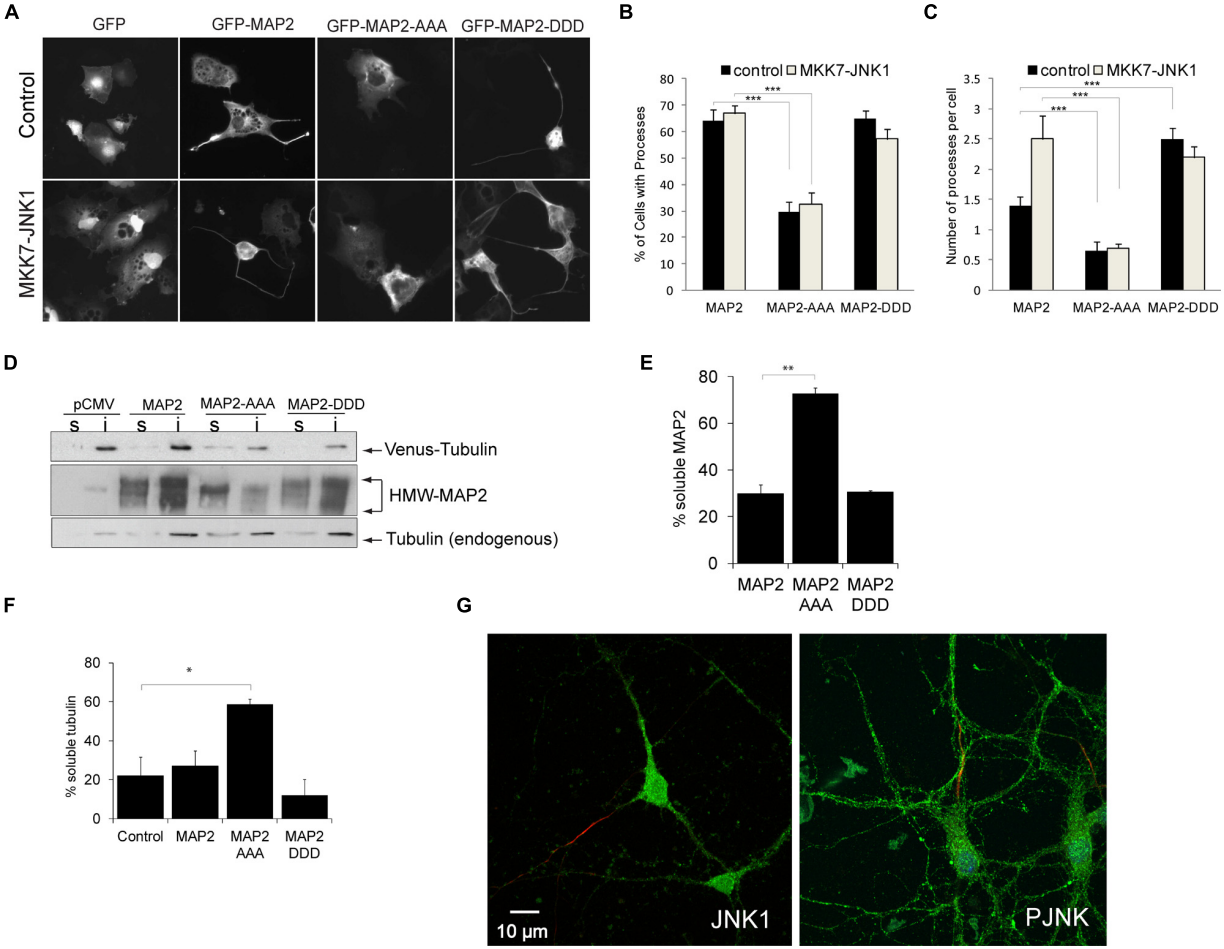
**PRD phosphorylation of MAP2 is necessary and sufficient for microtubule stabilization and MAP2-induced protrusion growth. (A)** COS-7 cell were transfected with GFP, GFP-MAP2, GFP-MAP2^T1619D,T1622D,T1625D^ (henceforth GFP-MAP2-DDD), or GFP-MAP2^T1619A,T1622A,T1625A^ (henceforth GFP-MAP2-AAA), with or without MKK7-JNK1 as shown. GFP-MAP2-induced protrusion growth while MKK7-JNK1 alone did not. **(B)** The proportion of cells that generated processes upon 48 h expression of MAP2 variant is shown. GFP-MAP2-AAA expressing cells failed to produce protrusions. **(C)** The number of processes per cell was counted. Expression of MKK7-JNK1 augmented the number of MAP2-generated processes. GFP-MAP2-DDD alone induced significantly more processes than did GFP-MAP2. **(D)** Fibroblasts transfected with Venus-tubulin together with GFP, GFP-MAP2, GFP-MAP2^T1619A,T1622A,T1625A^ (MAP2-AAA), or GFP-MAP2^T1619D,T1622D,T1625D^ (MAP2-DDD) were permeablized and the distribution of MAP2 or Venus-tubulin to the TX-100 soluble or insoluble fractions were quantified using immunoblotting. **(E)** Quantified data shows that GFP-MAP2^T1619A,T1622A,T1625A^ partitions to the soluble phase, while GFP-MAP2^T1619D,T1622D,T1625D^ remains in the TX-100 insoluble phase. **(F)** Quantitative data on Venus-tubulin stability. GFP-MAP2^T1619D,T1622D,T1625D^ stabilizes microtubules whereas GFP-MAP2^T1619A,T1622A,T1625A^ induces microtubule destabilization. **(G)** Polarized neurons at 12 days *in vitro* were immunostained for JNK1 (green) and ankyrin G (red; left panel) to highlight the axon, and P-JNK (green) and ankyrin G (red), right panel. JNK1 and P-JNK immunoreactivity was detected in both axonal and dendritic compartments. For all histograms, means ± SEMs are shown. Significance levels are in each instance compared to control conditions (GFP-MAP2). *p< 0.05; **p< 0.01; ***p< 0.005.

MAP2 binds laterally along microtubule polymers and confers rigidity to dendritic arbors (Felgner et al., 1997). To determine whether the JNK1 phosphorylated residues of HMW-MAP2 influence process formation, we prepared a phospho-mimicry mutant of HMW-MAP2 (GFP-HMW-MAP2^T1619D,T1622D,T1625D^), and expressed either this, GFP-HMW-MAP2^T1619A,T1622A,T1625A^ or GFP-HMW-MAP2-WT in COS-7 cells, which lack endogenous MAP2. Exogenous expression of GFP-HMW-MAP2 in COS-7 cells is sufficient to induce occasional protrusions from the soma (Björkblom et al., 2005; **Figures 2A,B**). However, expression of GFP-HMW-MAP2^T1619D,T1622D,T1625D^ led to a greater number of cells with protrusions (**Figures 2A,B**). Similarly, activation of JNK, increased the number of protrusions in GFP-HMW-MAP2^WT^-expressing cells (**Figure 2C**). Strikingly, cells that expressed GFP-HMW-MAP2^T1619A,T1622A,T1625A^ failed to generate protrusions altogether, even in the presence of active JNK1 (MKK7-JNK1; **Figures 2A–C**). Furthermore, GFP-HMW-MAP2^T1619A,T1622A,T1625A^ remained visibly soluble. These data indicate that JNK1-dependent phosphorylation of T1619, T1622, and T1625 of HMW-MAP2 is necessary and sufficient for protrusion growth.

### GFP-HMW-MAP2^**T1619D,T1622D,T1625D**^ CO-PURIFIES WITH MICROTUBULES GFP-HMW-MAP2^**T1619A,T1622A,T1625A**^ PARTITIONS TO THE SOLUBLE FRACTION THAT LACKS MICROTUBULES

Microtubules carry a net surface charge that is negative (Baker et al., 2001). Interestingly however, a generalized increase in phosphorylation of HMW-MAP2 was shown to increase HMW-MAP2 interaction with microtubules (Brugg and Matus, 1991). This interaction may be facilitated by small regions of positive charge that exist on the microtubule surface (Baker et al., 2001). To determine whether introduction of negative charges to T1619, T1622, and T1625 of HMW-MAP2 altered microtubule binding, we expressed GFP-HMW-MAP2^T1619D,T1622D,T1625D^, GFP-MAP2, or GFP-HMW-MAP2^T1619A,T1622A,T1625A^ in fibroblasts together with venus-tubulin, and measured partitioning along with microtubules to the TX100 insoluble fraction. GFP-MAP2^WT^ and GFP-HMW-MAP2^T1619D,T1622D,T1625D^ partitioned to the TX100-insoluble pellet with microtubules. However, GFP-HMW-MAP2^T1619A,T1622A,T1625A^ remained in the soluble fraction which lacked microtubules (**Figures 2D,E**). GFP-HMW-MAP2^T1619D,T1622D,T1625D^ increased the levels of polymerized tubulin (TX100-insoluble fraction) while GFP-HMW-MAP2^T1619A,T1622A,T1625A^ significantly reduced the percent of stable microtubules (**Figure 2F**). These data indicate that the JNK1 phosphorylation sites on HMW-MAP2 create a motif through which it can bind and stabilize microtubules.

HMW-MAP2 is specifically located in the dendritic compartment of neurons, yet physiologically active JNK is associated with axons (Oliva et al., 2006; Feltrin et al., 2012). We therefore examined the localization of JNK1 and active JNK (P-JNK) in polarized neurons. Axons were visualized using ankyrin G. We found prominent JNK1 immunoreactivity in the dendrites, furthermore P-JNK antibodies (which detect active JNK isoforms) revealed immunoreactivity throughout the dendritic compartment (**Figure 2G**). Thus JNK1 is present in dendrites where it is well positioned to phosphorylate HMW-MAP2 and alter dendrite structure.

### DENDRITE ARCHITECTURE IS ALTERED IN THE PRIMARY MOTOR CORTEX OF *Jnk1-/-* MICE

**FIGURE 3:**
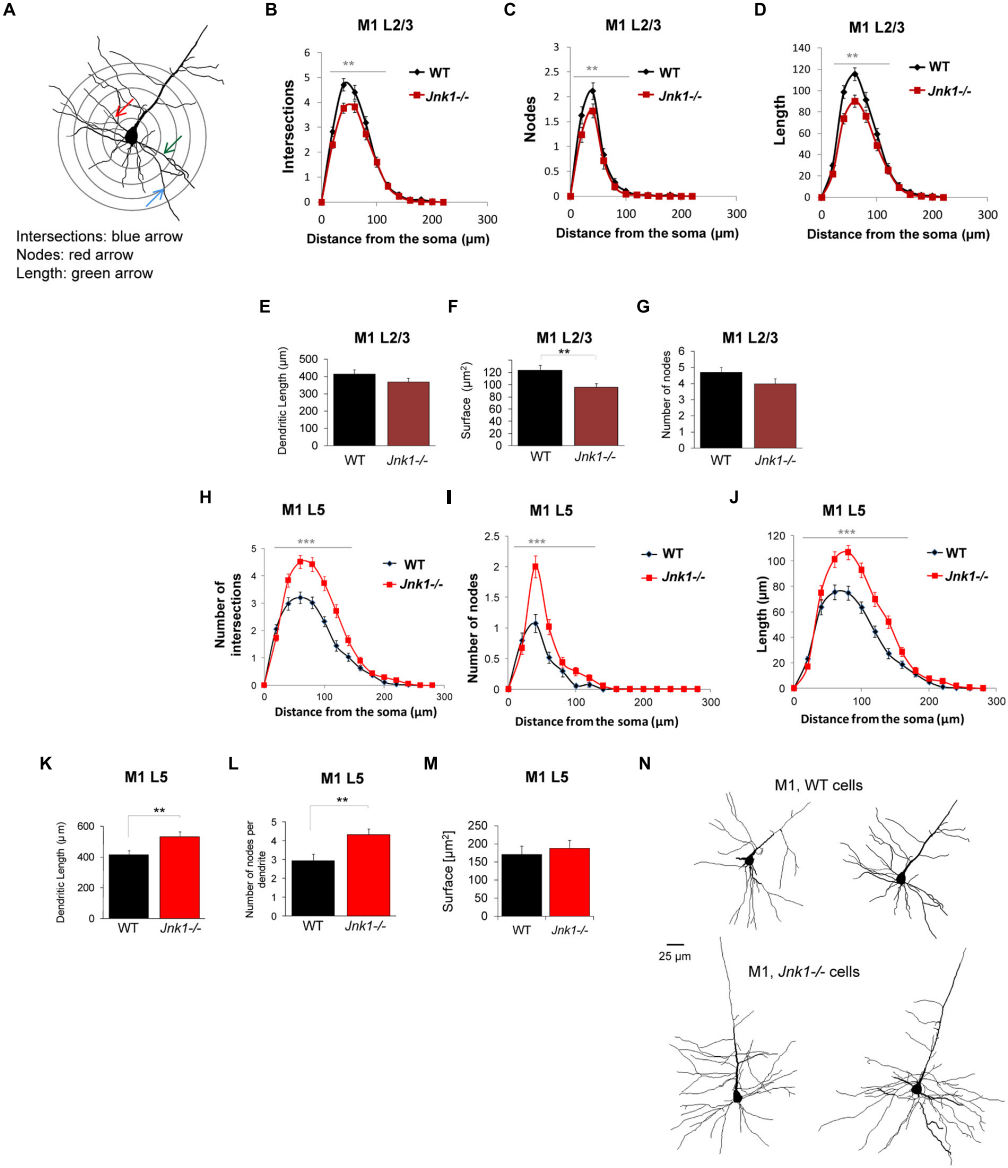
**Dendrite morphology is disrupted in the primary motor cortex of *Jnk1-/-* mice. (A)** Wild-type (WT) and *Jnk1-/-* mice at 6–8 months of age were loaded with Lucifer yellow dye and a Sholl analysis was carried out on single dendrites, measuring only basal dendrites from reconstructed three-dimensional images. **(B–D)** L2/3 neurons from the primary motor cortex (M1) were analyzed for dendrite complexity. The number of intersections, nodes, and length were calculated from single complete dendritic trees. There was a significant decrease in dendrite complexity in *Jnk1-/-* mice. **(E–G)** There was no significant change in the total dendrite length or number of nodes, though the total surface area was significantly decreased significantly in *Jnk1-/-* mice in L2/3 neurons. **(H–J)** In L5 neurons from M1, there was a significant increase in dendrite complexity exemplified as increased number of intersections, nodes and increased dendrite length in *Jnk1-/-* brains compared to wild-type. **(K–M)** The total dendritic tree length, number of nodes per tree and total surface area is shown. **(N)** Neurolucida tracings from M1 L5 neurons in WT and *Jnk1-/-* mice are shown. Means ± SEMs are indicated. For L2/3 neurons 81 WT and 61 *Jnk1-/-* dendritic trees were analyzed from a total of 67 cells. For L5 neurons, 49 WT and 63 *Jnk1-/-* trees were analyzed from a total of 47 cells. For **(B–D,H–J)**, significance was determined using one-way ANOVA. For **(E–G,K,L)**, significance was determined using Student’s *t*-test. 0.001.

To evaluate the effect of JNK1 on dendrite architecture *in vivo*, we loaded neurons in brain slices from WT and *Jnk1-/-* mice with lucifer yellow dye. Many of the pyramidal neurons we traced in L5 had large somas consistent with their being corticospinal projecting neurons of L5B (Oswald et al., 2013). Three-dimensional confocal sections underwent Sholl analysis. Concentric rings were spaced 20 μm apart centered around the soma (as illustrated **Figure 3A**). Intersections, nodes and length of dendritic arbors were mapped within each concentric ring. Only basal dendrites were measured, as dye loading was frequently incomplete in the apical dendrites and tufts, which were excluded. We started with layer 2/3 (L2/3) neurons in the rostral and caudal forelimb areas (RFA, CFA), medial agranular cortex AGm, and lateral agranular cortex (AGl) of the mouse primary motor cortex (M1; Tennant et al., 2011). Small and medium sized pyramidal cells were included. There was reduced complexity in the upper layer neurons of *Jnk1-/-* mice, defined as fewer intersections, a smaller number of nodes, and reduced dendritic length (**Figures 3B–G**). In contrast, the layer 5 (L5) neurons of *Jnk1-/-* mice showed increased dendrite complexity (**Figures 3H–L,N**). Moreover, the extent of architecture alterations in the large pyramidal neurons of L5 was greater than that observed in the upper L2/3. Representative tracings of primary motor cortex neurons from the deeper layers are shown (**Figure 3N**). The increase in dendrite complexity in L5 neurons may have developed at least in part, to offset the reduced input from L2/3 neurons which display reduced dendrite elaboration. The overall elaboration of dendrites observed in the projection neurons of the primary motor cortex in these mice are consistent with our previous findings in isolated cerebellar granule neurons from *Jnk1-/-* mice (Björkblom et al., 2005), suggesting that a common mechanism exists.

### EXPRESSION OF CAG-EGFP-HMW-MAP2^**T1619D,T1622D,T1625D**^ *IN VIVO* IN THE MOTOR CORTEX ALTERS THE ARCHITECTURE OF L2/3 DENDRITES

**FIGURE 4:**
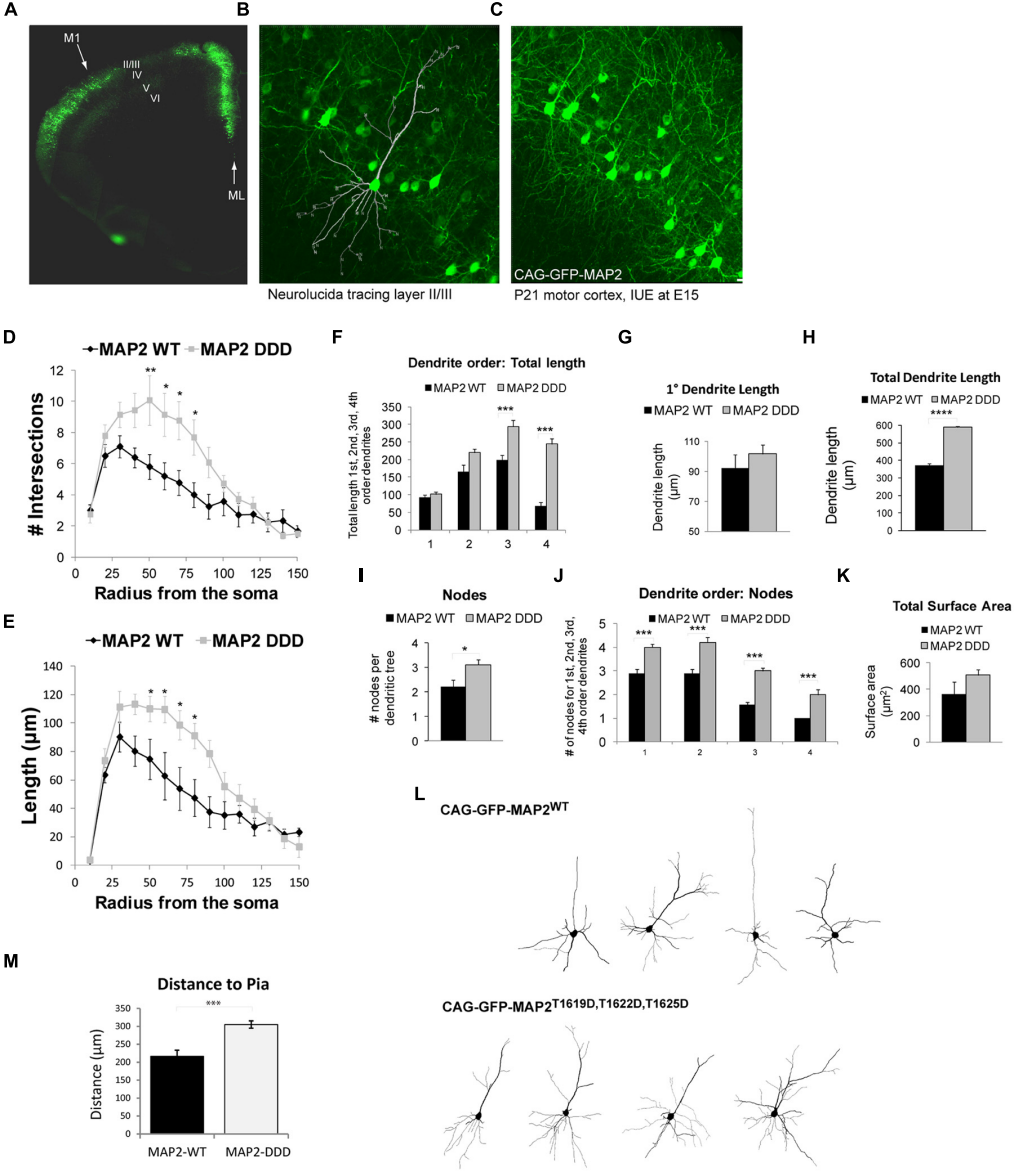
**MAP2^**T1619D,T1622D,T1625D**^ regulates basal dendrite architecture *in vivo***. **(A)** Mice electroporated with DNA at E15 *in utero* were sacrificed at postnatal day 21. The fluorescent micrograph of a cerebral hemisphere shows expression of GFP in L2/3 of Motor cortex 1 (M1). The midline (ML) is indicated. **(B,C)** Higher magnification confocal micrographs of L2/3 depicting the neurolucida tracing **(B)** and the dense network of GFP-expressing cells **(C)**. **(D,E)** The number of intersections **(D)** and dendrite length **(E)** from Sholl analysis of whole cell basal dendrites from mice electroporated *in utero* with pEGFP-CAG-MAP2^T1619D,T1622D,T1625D^ revealed increased dendrite complexity compared to mice expressing pEGFP-CAG-MAP2^WT^. **(F)** There was a significant increase in dendrite length in third and fourth order branches. **(G)** Primary dendrite length however remained unchanged. **(H)** There was a significant increase in total dendrite length in MAP2^T1619D,T1622D,T1625D^ expressing mice. **(I)** The average number of nodes per dendritic tree was increased in *Jnk1-/-* mice. **(J)** The number of nodes was significantly increased at every dendrite order. **(K)** The surface area measurements from neurons in GFP-MAP2^WT^ or GFP-MAP2^T1619D,T1622D,T1625D^ expressing mice are shown. **(L)** Representative Sholl traces from mice expressing GFP-MAP2^WT^ or GFP-MAP2^T1619D,T1622D,T1625D^ are shown. Statistical analysis in **(D–F,J)** were two-way ANOVA with post hoc Bonferroni test of significance for individual points. *p < 0.05; **p< 0.01; ***p< 0.005. **(G–I,K,M)** were analyzed by Student’s *t*-test. *p< 0.05; ***p< 0.001; ****p< 0.0001. Twenty-four dendritic trees were analyzed from four separate mice. (Ten from WT and 14 from GFP-^T1619D,T1622D,T1625D^–expressing mice. **(M)** The distance from the pia of neurons expressing GFP-MAP2^WT^ or GFP-MAP2^T1619D,T1622D,T1625D^ is indicated. Neurons expressing GFP-MAP2^T1619D,T1622D,T1625D^ remained further from the pial surface in 21d mice.

Pyramidal neuron dendrites of the motor cortex respond to external cues and genetic programs to undergo arborization phases from P7 to P21 (Aizawa et al., 2004; Urbanska et al., 2008; Romand et al., 2011). We showed that GFP-HMW-MAP2^T1619D,T1622D,T1625D^ facilitates protrusion growth in COS7 cells. To determine if phosphorylation of MAP2 on these sites altered dendrite architecture *in vivo,* we prepared HMW-MAP2 phosphorylation site mutants under the control of the CAG promoter and introduced them to the developing motor cortex using targeted electroporation of E15 embryos. This resulted in efficient labeling of cells in L2/3 of the motor cortex (**Figure 4A**), however as this technique labels cells destined for the superficial layers, L5 cells did not express ectopic GFP-HMW-MAP2. Dendrite architecture in L2/3 was analyzed from the mice when they reached postnatal day 21, by which time the basal dendrites have developed most of their adult features (Romand et al., 2011). Basal dendrites were analyzed in entirety for each cell as outlined (**Figures 4A–C**). Neurons expressing GFP-HMW-MAP2^WT^ displayed a similar architecture to L2/3 motor cortex neurons labeled with Lucifer yellow, with no alteration in total dendrite length (**Figure 3E** compared to **Figure 4H**), although the first branching occurred closer to the soma in these cells (**Figures 4D** and **Figure 3B**). Interestingly however, GFP-HMW-MAP2^T1619D,T1622D,T1625D^ expressing L2/3 cells produced significantly more elaborate dendrites, with increased intersections (**Figure 4D**). This large increase in the number of intersections resembles the arborization pattern observed in more mature neurons (**Figure 3D**; Romand et al., 2011).

Higher order dendrite length (third and fourth order) was considerably increased in motor cortex cells expressing GFP-HMW-MAP2^T1619D,T1622D,T1625D^ (**Figure 4E**), although the primary dendrite length remained unchanged (**Figures 4F,G**). Extensive arbor branching, increasing significantly from the primary to the fourth order dendrites (**Figures 4I,J**), was visible in GFP-HMW-MAP2^T1619D,T1622D,T1625D^-expressing cells, and there was a trend towards increased total surface area (**Figure 4K**). Representative images of L2/3 pyramidal neurons labeled with GFP-HMW-MAP2^WT^ or with GFP-HMW-MAP2^T1619D,T1622D,T1625D^ are shown (**Figure 4L**). In summary, the overall effect of GFP-HMW-MAP2^T1619D,T1622D,T1625D^ expression compared to GFP-MAP2^WT^ was to increase the length of higher order dendrites and to increase branching of dendrites at all orders. The overall increase in total dendrite length was 50%, partially explained by the extensive increase in branching (**Figure 4H**). It is notable that neurons expressing GFP-HMW-MAP2^T1619D,T1622D,T1625D^ resided a greater distance from the pial surface than neurons expressing GFP-HMW-MAP2^WT^ (**Figure 4M**). As previously shown, cytosolic JNK1 acts as a negative regulator that retards radial migration (Westerlund et al., 2011). HMW-MAP2 phosphorylation by JNK1 may thus prematurely halt the migration.

### CELL SOMA AREA IS INCREASED IN L5 M1 PYRAMIDAL NEURONS IN *Jnk1-/-* MICE

**FIGURE 5:**
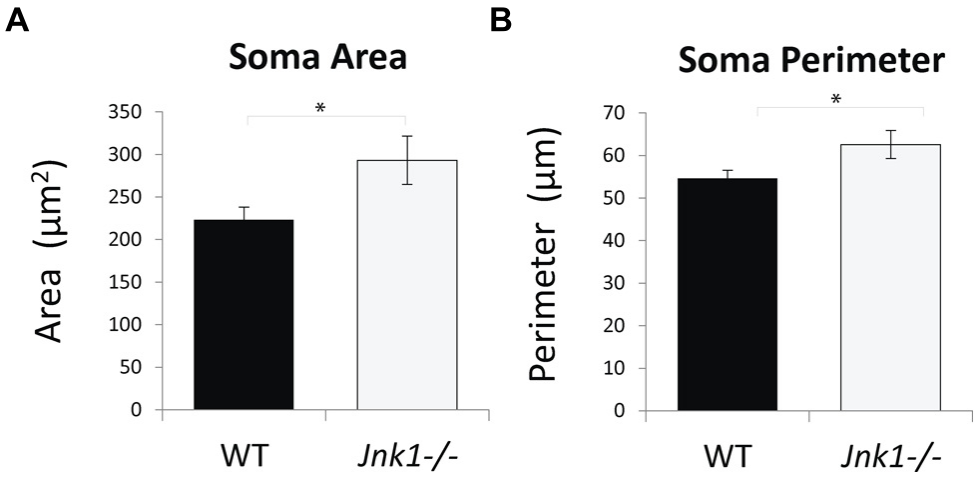
**Cell soma size is increased in M1 pyramidal neurons of *Jnk1-/-* mice. (A,B)** Cell soma areas and perimeters from Lucifer yellow-loaded neurons in L5 of M1 from wild-type and *Jnk1-/-* mice are shown. There was a significant increase in soma size in *Jnk1-/-* mice. Six wild-type and 13 *Jnk1-/-* cells were counted from different animals. Means ± SEMs are indicated. *p< 0.05.

Examination of cell soma size in M1 L5 neurons indicated that somas were on average 35% larger in *Jnk1-/-* mice (**Figures 5A,B**). This was done by stereological measurement of cell soma cross sectional area and perimeter from three-dimensional images of lucifer yellow loaded pyramidal neurons. The increased soma size observed in Jnk1*-*/*-* pyramidal neurons is consistent with increased dendritic load in L5 (**Figure 3**), as soma size has been shown to correlate with dendritic material load (van Pelt et al., 1996).

### *Jnk1-/-* MICE SHOW DEFECTIVE MOTOR SKILLS

**FIGURE 6:**
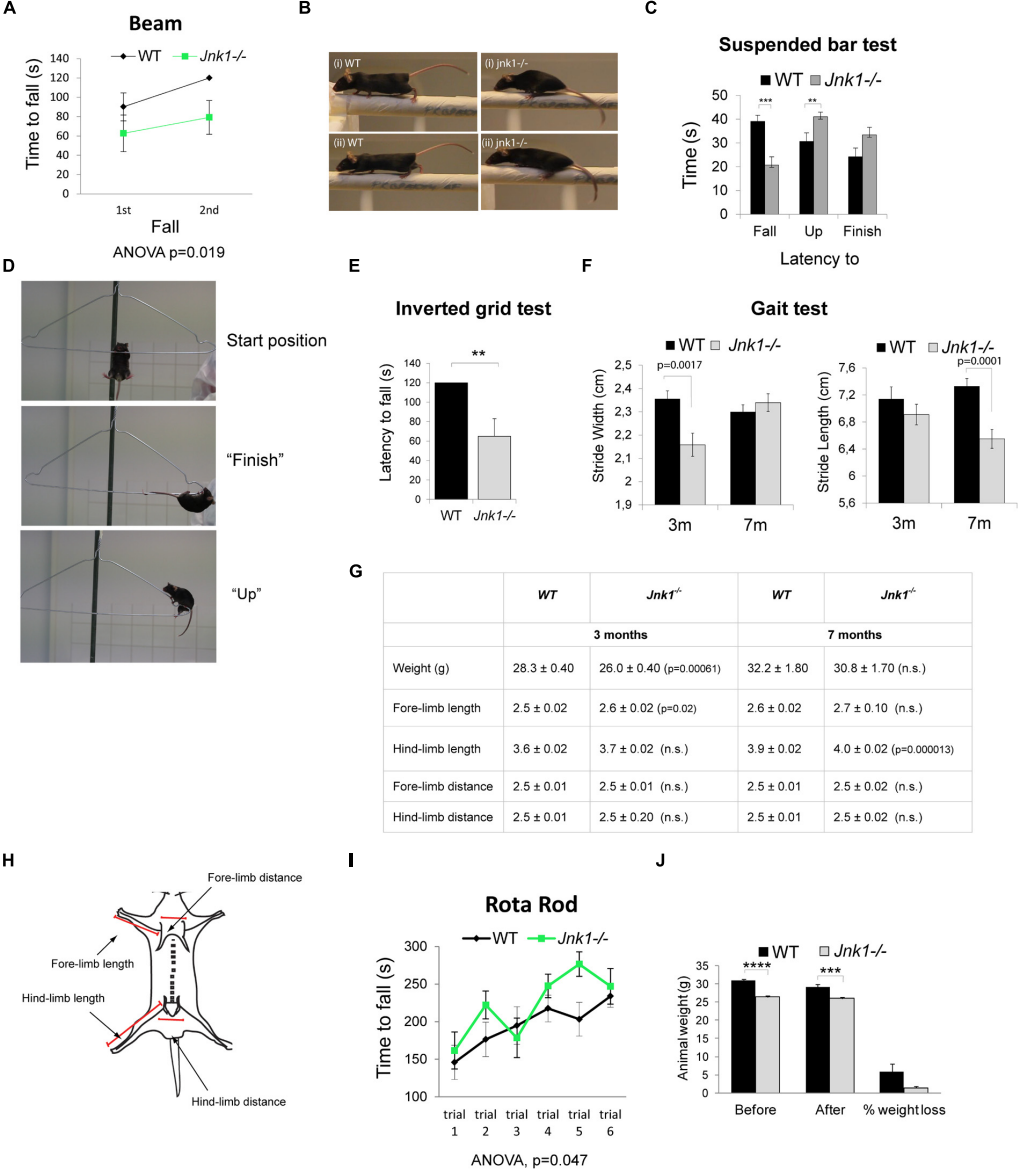
***Jnk1-/-* mice show impaired motor skills. (A)** Wild-type (WT) and *Jnk1-/-* mice were subjected to the beam walking test. Two trials were undertaken and time to fall measured. In the second trial *Jnk1-/-* mice showed a shorter latency to fall than WT mice. Eight male mice of each genotype were tested. **(B)** *Jnk1-/-* mouse paws often slipped to the side in the beam test. WT mice did not show this behavior. Representative images from four individual mice are shown. **(C)** To determine muscle and grip strength, mice were subjected to a grip test using a suspended wire. The results show the latency to “fall,” time to “finish,” and time to “up” (reach 5 cm on the diagonal bar with the forepaws) as illustrated. *Jnk1-/-* mice showed a significantly shorter latency to fall than WT. Furthermore, 57% of *Jnk1-/-* mice failed to complete the course compared to only 29% of WT mice. Seventeen male WT and 21 male *Jnk1-/-* mice were tested. **(D)** Representative images of mice in the “start,” “finish,” and “up” positions. **(E)** Fore limb and hind limb strength was measured using the latency to fall of mice hanging on an inverted grid. Five WT and 5 *Jnk1-/-* mice were measured. **(F)** Gait was assessed by measuring distances between footprints from 3 month (3 m) and 7 month (7 m) mice. *Jnk1-/-* mice at 3 m displayed a significantly narrower stride width than WT mice. By 7 m the difference in stride width was no longer significant however stride length was significantly reduced in *Jnk1-/-* mice. Twelve mice from each genotype were analyzed. **(G)** A table displaying the anatomical measurements of WT and *Jnk1-/-* mice at 3 m and 7 m is shown. Meaned data ± SEM from 11 WT and 12 *Jnk1-/-* mice at 3 m and from five WT and seven *Jnk1-/-* mice at 7 m, are shown. Where significant difference to WT exists, *p-*values are indicated. N.s. indicates that differences compared to controls were not significant. **(H)** The scheme illustrates the measurement strategy used to determine length of forelimbs and hindlimbs. **(I)** Mice were subjected to the accelerating rotarod test. There was no phenotypic difference between genotypes. Data shown are meaned from eight WT and eight *Jnk1-/-* male mice. **(J)** The average weight of 8-week old WT and *Jnk1-/-* mice before and after the battery of behavioral tests is shown. *Jnk1-/-* mice were significantly lighter than WT. For all graphs, means ± SEMs are shown. Levels of significance determined by ANOVA or Student’s *t*-test, are as follows: **p< 0.01; ***p< 0.001; ****p< 0.0005.

Since we observed dendrite complexity alterations in L2/3 and L5 of the primary motor cortex, which controls complex muscle activation patterns (Levine et al., 2012), we tested the performance of *Jnk1-/-* mice using a battery of behavioral tests assessing motor coordination and strength. These tests require a high degree of motor function and relatively subtle deficits can be revealed. We first tested motor activity and balance of mice on the raised beam. *Jnk1-/-* mice performed poorly while traversing the beam, showing a significantly reduced latency to fall compared to wild-type mice (**Figure 6A**). Notably, knockout mice frequently had difficulty with placement of hind paws and forward movement of hind limbs (**Figure 6B**), indicating impaired coordination and balance. We next monitored motor coordination and grip strength by measuring the latency to fall from a suspended wire (Lalonde et al., 1992; Baldo et al., 2012). Mice were placed hanging by their two forelimbs from a wire coat-hanger. Mice lacking *Jnk1* showed a reduced latency to fall from the wire suggesting weakened grip strength (**Figures 6C,D**). When they did complete the task, *Jnk1-/-* mice took more time to do so (**Figure 6C**). During the 45 s time allocated, 57% of *Jnk1-/-* mice failed to complete the task compared to 29% of wild-type mice. We also measured grip strength using the inverted grid test where both forelimbs and hindlimbs are used to grip to an inverted grid. Mice lacking *Jnk1* showed significantly reduced latency to fall compared to wild-type (**Figure 6E**). Together these data suggest impaired motor coordination and strength in mice lacking *Jnk1*.

A more detailed examination of motor coordination and synchrony is provided by measuring gait using the footprint test (Brooks and Dunnett, 2009). Manual tracing of footprints revealed a reduction in stride width in 3 month old *jnk1-/-* mice compared to wild-type (**Figure 6F**). This difference between genotypes was rectified by 7 months. Interestingly though, in 7 month old knockout mice, the stride length was significantly reduced. While we detected a minor (2%) increase in hindlimb length in older knockout mice, this seems unlikely to account for the more significant (15%) decrease in stride length (**Figures 6F,G**). The small difference in forelimb and hindlimb lengths may be explained by osteoclast deficits as JNK1 regulates osteoclast differentiation (David et al., 2002), though no bone defects have been reported in *Jnk1-/-* mice. Finally, we measured ventral length, but found no differences (not shown).

We also subjected mice to the rotarod test (**Figure 6I**). There was no phenotypic difference between wild-type and knockout mice when they were placed on an accelerating rod, suggesting that gross motor skills were intact. Similarly, *Jnk1-/-* mice showed no locomotive defect in the open field test (not shown). The rotarod test is considered a less sensitive test of coordination (Brooks and Dunnett, 2009), with the drawback that heavy mice perform poorly compared to lighter mice. As the *Jnk1-/-* mice are 14.3 ± 1.7% lighter than wild-type mice (**Figure 6J**), it is possible that an offset due to reduced body weight masks an underlying deficit in coordination and balance control. Of significance, the footprint test of gait is one of the few tests that translates directly from animal to human studies (Brooks and Dunnett, 2009). It is worth commenting that we did not observe overt abnormalities with these mice while under observation in their home cages, for example there were no signs of tremors or explicit walking or running behaviors. This is the first report of motor behavior disturbance *Jnk1-/-* mice, though in *Caenorhabditis elegans*, JNK-1 regulates coordinated body movement (Villanueva et al., 2001). In summary, phenotypic differences emerged in tests that required skilled coordination of limb movement, where *Jnk1-/-* mice exhibited significantly impaired behavior.

## DISCUSSION

In this study, we show using three-dimensional Sholl analysis of lucifer yellow-loaded cells that the dendritic fields of L2/3 and L5 pyramidal neurons in the primary motor cortex are significantly altered in *Jnk1-/-* mice. To understand the mechanism, we studied the JNK1 substrate HMW-MAP2, a microtubule stabilizing protein that specifically localizes to dendrites. We demonstrate that JNK1 phosphorylates three sites in the COOH-terminal domain of HMW-MAP2 *in vitro* and in brain, and show that this phosphorylation is necessary and sufficient to stabilize microtubules in isolated cells, while ectopic expression of pseudophosphorylated GFP-HMW-MAP2 *in vivo* in the motor cortex mimics JNK1 dendritic arborization changes. Together these findings indicate that JNK1 phosphorylation of HMW-MAP2 on these residues alters the dendritic field of neurons *in vivo*. Consistent with this, we report functional deficits in motor coordination and balance in *Jnk1-/-* mice undergoing a battery of behavioral tests. Together these results highlight the importance of JNK1 in regulating dendrite architecture in the cortex and in determining motor skill behavior.

Microtubule associated proteins (MAPs) are enriched in the nervous system where they confer stability to microtubules by inhibiting a state of dynamic instability that is characteristic of non-neuronal cells (Sanchez et al., 2000). HMW-MAP2 is a 280 kDa MAP that imparts dendritic identity (Harada et al., 2002; Farah and Leclerc, 2008). It crosslinks microtubule protofilaments, facilitated by its C-terminal PRD (Hirokawa et al., 1996; Sanchez et al., 2000; Al-Bassam et al., 2002). While HMW-MAP2 is a highly phosphorylated protein, the outcome of site-specific phosphorylation has not been previously tested either *in vitro* or *in vivo*. Our data indicates that JNK1 phosphorylates T1617, T1620, and T1623 (equivalent to T1619, T1622, and T1625 in rat HMW-MAP2) in the cortex and hippocampus of early postnatal mice, as phosphorylation on these sites is reduced in *Jnk1-/-* mice. Site-directed mutagenesis indicates that the functional consequence of this phosphorylation is to switch HMW-MAP2 from a form that interacts poorly with microtubules and fails to support process growth, to a form that binds avidly and promotes outgrowth (**Figures 1** and **Figure 2**).

*In vivo*, we show that ectopic expression of GFP-HMW-MAP2^T1619D,T1622D,T1625D^ (phospho-mimicry form) in L2/3 of M1, substantially increases dendrite arborization (**Figures 4D,I,J**). This is likely to be physiologically meaningful because (i) these sites are phosphorylated *in vivo* in brain (**Figures 1C–G**) and (ii) phosphorylation of this domain increases during development and correlates with increased branching of cultured neurons (Díez-Guerra and Avila, 1993). Moreover, ectopic expression of pseudo-phosphorylated HMW-MAP2 augments arbor length, consistent with earlier findings in *MAP2* knockout mice where arbor length was reduced (Harada et al., 2002). The extensive increase in higher order dendrite branching upon ectopic expression of phospho-mimicry HMW-MAP2 is somewhat unexpected. However, new arbor formation requires coordinated interplay between microtubules and actin (Farah and Leclerc, 2008), and MAP2 is capable of binding and crosslinking actin, and co-localizes with actin at sites of protrusion sprouting (Dehmelt et al., 2003; Roger et al., 2004). Thus, this phosphorylation may provide a switch whereby arborization of dendrites can be regulated. For example, the pseudo-phosphorylated state of GFP-HMW-MAP2 may protect those dendrites from pruning that otherwise occurs between P7 and P21 (Aizawa et al., 2004; Urbanska et al., 2008; Romand et al., 2011). Together these results indicate that MAP2 mediates JNK1 regulation of dendrite morphology in L2/3 of the motor cortex.

Receptive fields of dendritic arbors determine where, and to what extent the neuron receives synaptic input. In contrast to L2/3, JNK1 substantially decreases the total length and branching of basal dendrites in L5 (by 20–30%, **Figures 3H–L**), while only moderately increasing the dendritic field in L2/3 (**Figures 3B–G**). The increased dendritic field in L5 of *Jnk1-/-* mice may result at least partly from the decreased L2/3 input (**Figures 3B–G), as L2/3 provides the foremost descending input to L5 (Young and Kaas, 2012**; Kaneko, 2013). However, the opposing effect of JNK1 on dendrite architecture in L2/3 and L5 may be due differing cellular contexts. Thus, whether MAP2 phosphorylation by JNK1 exerts increased dendrite complexity (as occurs in L2/3), or decreased complexity (as in L5), may depend on the JNK1 substrate composition of the neurons in defined layers. Indeed, JNK1 phosphorylates the microtubule regulatory protein SCG10 (Tararuk et al., 2006), which directly controls dendrite morphology (Ohkawa et al., 2007). Also, JNK1 phosphorylates the actin bundling protein MARCKSL1 and several proteins regulating synaptic activity that can indirectly influence dendrite shape, e.g., PSD95 (Kim et al., 2007), GluR2, and GluR4 AMPAR subunits (Thomas et al., 2008). Regardless of how the phosphorylation of multiple JNK1 targets cooperate to influence the final outcome *in vivo*, the control of receptive fields in L2/3 and L5 by JNK1 will likely determine the excitatory and inhibitory balance in M1, a region that is centrally involved in controlling the coordination and execution of movement.

Consistent with our finding that JNK1 regulates dendritic field, JNK1 is activated by signaling through the Down syndrome cell adhesion molecule (DSCAM) receptor (Qu et al., 2013), a key regulator of dendritic self-avoidance (Zipursky and Grueber, 2013). Thus, JNK1 may mediate DSCAM signals to the cytoskeleton via substrates such as HMW-MAP2, to control territorial coverage of dendrites. Furthermore, JNK1 is activated by cues that are known to regulate dendrite morphogenesis including netrin, semaphorin-3A, and BMPRII (de Anda et al., 2012; Qu et al., 2013).

It is expected that the structural control elicited by JNK1 in L2/3 and L5 will influence the information flow of the cortex, as sub-cortical projections from L5 pyramidal neurons govern voluntary movement and stereotypical behavior in the areas that control forelimb and hindlimb function (Tennant et al., 2011; Levine et al., 2012). Consistent with this, we observe relevant behavioral deficits in *Jnk1-/-* mice undergoing tasks that involve coordination (beam, suspended wire), balance (beam), and strength (suspended wire and inverted grid; **Figure 6**). On the beam, *Jnk1-/-* mice exhibited impaired hindlimb placement, gripping the beam from the sides instead of placing their paws on the upper surface. Appropriate limb placement requires a reflex to tactile stimulation that is mediated by the motor cortex (Metz and Whishaw, 2002), as well as motor coordination (Young and Kaas, 2012). Analysis of gait revealed that in older mice lacking *Jnk1*, stride length was significantly decreased (**Figure 6F**). Aside from irregularities in M1, these behaviors could in principle result from progressive decline in muscle tone, or from degeneration of cerebellar input. There is a pretext for a cerebellar component, as targeted deletion of *Jnks 1, 2*, and *3* (Xu et al., 2011) or knockout of *Jnk1* (Björkblom et al., 2005; Xu et al., 2011), alters Purkinje neuron dendrite architecture. Moreover, altered stride width, a classic hallmark of cerebellar dysfunction (Gilman et al., 1981), is reduced in young adult mice lacking *Jnk1-/-*.

Interestingly though, this difference in stride width was no longer detectable in older mice (**Figure 6F**), and other hallmarks of cerebellar dysfunction, such as tremor activity, or difficulty with repeated movements (Gilman et al., 1994), were not detected during passive observation of the mice. Possible differences in corticospinal tract axonal projections (Martin, 2005), which we have not examined here, may also influence these behaviors. Nonetheless, micro-stimulation of deep L5 neurons in M1 induces somatotopically mapped movements (Tennant et al., 2011), so it is possible that the dendritic field alterations observed contribute to the deficits in motor behavior.

Genetic anomalies at several levels of the JNK cascade confer susceptibility to psychiatric disorders (Weiss et al., 2008; de Anda et al., 2012; Winchester et al., 2012; Kunde et al., 2013; Coffey, 2014), indicating that disturbance of the JNK pathway may be central to the pathology. Among these, schizophrenia and autism stand out as dendrite disorders that are accompanied by motor deficits. Schizophrenia is associated with gray matter loss during development (Bennett, 2011). In particular, basal dendrite complexity is reduced in L5 neurons and MAP2 levels are lowered (Broadbelt et al., 2002). The regression of synapses that accompanies dendrite reduction leads to re-organization of topographical maps (Bennett, 2011), and motor deficits are present in 66% of first-episode, never medicated patients, and in 80% of chronically medicated patients (Walther and Strik, 2012). Moreover, decreased soma size has been reported in schizophrenia patients (Rajkowska et al., 1998). We show that JNK1 regulates each of these disease hallmarks. Thus, JNK1 constrains soma size (**Figure 5**) and dendritic field complexity (**Figures 3H–N**), while at the same time playing a requisite role in controlling motor skills (**Figure 6**). Motor and dendrite anomalies are typical for autistic disorders (Zoghbi, 2003; Snow et al., 2008; Jiang et al., 2013). TAOK2 is a MAP3K that is located in a region of chromosome 16p11.2 carrying susceptibility to autism (Weiss et al., 2008). Knockdown of *Taok2* reduces basal JNK1 activity and decreases dendrite arborization in L2/3 of the cortex (de Anda et al., 2012), in agreement with our findings in *Jnk1-/-* mice (**Figure 3**).

Irregular growth or maintenance of dendrites, contributes to the development of psychiatric disease (Jan and Jan, 2010). Given that increased risk for these disorders is associated with genetic abnormalities in JNK signaling (Coffey, 2014), our findings in *Jnk1-/-* mice are important as they demonstrate the molecular and behavioral consequences of interfering with JNK1 pathway signaling and provide grounds for improved understanding of the molecular underpinnings of psychiatric disorders.

## Conflict of Interest Statement

The authors declare that the research was conducted in the absence of any commercial or financial relationships that could be construed as a potential conflict of interest.
